# Incidence and influencing factors of tooth discoloration in children using doxycycline: a meta-analysis

**DOI:** 10.3389/fped.2025.1644231

**Published:** 2025-08-22

**Authors:** Kun Ma, Mingjing Lu, Hao Li, Xin Yuan, Ying Zhang, Qiuying Ni, Yun Li, Xiaolin Dong, Jingjing Guo

**Affiliations:** ^1^ Department of Pediatrics, The First Afﬁliated Hospital of Shandong First Medical University & Shandong Provincial Qianfoshan Hospital, Jinan, China; ^2^ Qilu Institute of Technology, Jinan, China; ^3^ Jinan Central Hospital, Cheeloo College of Medicine, Shandong University, Jinan, China; ^4^ Department of General Medicine, Central Hospital Affiliated to Shandong First Medical University, Jinan, China

**Keywords:** doxycycline, children, tooth discoloration, adverse events, meta-analysis

## Abstract

Doxycycline is traditionally avoided in children under 8 years due to concerns about permanent tooth discoloration. This meta-analysis aimed to assess the incidence of tooth discoloration in children treated with doxycycline and identify influencing factors. A systematic search of PubMed, Embase, Web of Science, Wanfang, and CNKI was conducted up to January 14, 2025, for studies reporting tooth discoloration in children (<18 years) treated with doxycycline. Pooled incidence was estimated using a random-effects model. Subgroup and univariate meta-regression analyses explored potential modifiers. Seventeen studies comprising 1,025 children were included. The pooled incidence of tooth discoloration was 0.92% (95% CI: 0.34%–1.50%) with no significant heterogeneity (I² < 0%). Subgroup analyses showed no significant differences by region, study design, age group, administration route, or assessment method. Meta-regression indicated no significant effects from publication year, sample size, mean age, dose, treatment duration, follow-up length, or study quality. The incidence of tooth discoloration following doxycycline use in children is low. These findings support the potential safety of doxycycline in pediatric populations, including those under 8 years of age, when clinically warranted.

**Systematic Review Registration:**

PROSPERO (CRD420251009690).

## Introduction

Doxycycline is a second-generation, broad-spectrum, semi-synthetic tetracycline antibiotic derived from oxytetracycline (1, 2). It exhibits a high degree of lipophilicity, a long plasma half-life, and excellent oral bioavailability (3). Doxycycline acts primarily by binding to the 30S ribosomal subunit of bacteria, inhibiting protein synthesis and thereby exerting a bacteriostatic effect (4). Its favorable pharmacokinetics, including good tissue penetration and a lower affinity for calcium than that of earlier tetracyclines, make it a valuable agent for treating a wide range of infections, including respiratory tract infections, atypical pneumonia, rickettsial diseases, malaria (prophylaxis), sexually transmitted infections, and inflammatory conditions such as acne and rosacea (3, 5, 6).

Despite its therapeutic advantages, the use of doxycycline in children—particularly those under 8 years of age—has historically been limited due to concerns about adverse effects on developing teeth (7, 8). Tetracyclines, including doxycycline, are known to bind to calcium ions and can become incorporated into the hydroxyapatite matrix of teeth (9) and bones (10) during mineralization. This chelation process can lead to permanent tooth discoloration, typically presenting as yellow, brown, or gray staining, and, in severe cases, enamel hypoplasia (11). The mechanism is believed to involve oxidation of the tetracycline-calcium orthophosphate complex upon light exposure, resulting in pigmentation (12). Such discoloration is not only irreversible but may also lead to psychosocial distress and reduced quality of life, particularly during adolescence and adulthood (12).

While the association between tetracycline use and dental staining is well-documented, the risk specifically associated with doxycycline has been increasingly questioned. Doxycycline has a lower calcium-binding capacity than that of first-generation tetracyclines, and recent clinical observations suggest that the incidence of tooth discoloration may be much lower than previously assumed, especially when the antibiotic is used for short durations (8). In recognition of its effectiveness in life-threatening infections such as Rocky Mountain spotted fever, current guidelines from the Centers for Disease Control and Prevention (CDC) support the use of doxycycline in young children, including those under 8 years, when the benefits outweigh the risks (13). However, regulatory labeling and clinical practice in many regions remain cautious, often discouraging its use in children under 8 years of age. Given this clinical uncertainty, a comprehensive synthesis of the available evidence is warranted. Therefore, in this study, we performed a meta-analysis to estimate the overall incidence of tooth discoloration in children treated with doxycycline and to examine potential influencing factors through subgroup and meta-regression analyses.

## Methods

This meta-analysis followed the PRISMA 2020 guidelines (14, 15) and the Cochrane Handbook for Systematic Reviews and Meta-Analyses (16) for protocol design, data extraction, statistical analysis, and results reporting. The protocol of the meta-analysis has been registered at PROSPERO with the identifier CRD420251009690.

### Literature search

Relevant studies for this meta-analysis were identified through a comprehensive search of the PubMed, Embase, Web of Science, Wanfang, and China National Knowledge Infrastructure (CNKI) databases using a broad range of search terms, which included: (1) “doxycycline” OR “tetracycline”; (2) “tooth” OR “teeth” OR “dental”; (3) “staining” OR “discoloration” OR “pigmentation” OR “events”; and (4) “child” OR “children” OR “pediatric” OR “adolescents”. The search was limited to human studies and full-length articles published in English or Chinese in peer-reviewed journals. Additionally, references from relevant original and review articles were manually screened for further eligible studies. The search span was from database inception to January 14, 2025. The detailed search strategy for each database is shown in Supplementary File 1.

### Inclusion and exclusion criteria

The eligibility criteria for studies were established based on the PICOS framework:

P (patients): Children (defined as <18 years of age) receiving doxycycline treatment, with no restrictions on sex or ethnicity. The study population included children treated with doxycycline for infectious diseases and other indications. Although enamel mineralization is generally complete by 8 years of age, studies involving older children were included to assess any delayed or previously unrecognized discoloration, as some late-forming teeth (e.g., second molars) may still be affected.

I (exposure): Doxycycline treatment, regardless of dosage, route of administration (e.g., oral or injection), and duration of treatment.

C (control): As this review focused on single-arm clinical studies, no direct comparator group was included.

O (outcome): The outcomes of this study focused on the incidence of tooth discoloration in children who received doxycycline. The evaluation methods and diagnosis of tooth discoloration were consistent with the criteria of the original studies. Both evaluations by pediatric dentists and other validated methods, including self-report and clinical observation, were accepted.

S (study design): The studies were comprised of longitudinal observational studies, including cohort studies, nested case-control studies, and *post-hoc* analyses of clinical studies.

Comparative clinical studies were also included if the incidence of tooth discoloration in the pediatric patients allocated to the doxycycline arm was adequately reported.

Studies were excluded if they were reviews, editorials, meta-analyses, preclinical research, included adult patients, were not limited to pediatric patients using doxycycline, or did not report the incidence of tooth discoloration. When population overlap occurred, the study with the largest sample size was selected for inclusion in the meta-analysis.

### Study quality assessment and data extraction

Two authors independently conducted the literature search, study selection, quality assessment, and data extraction, resolving discrepancies through discussion with the corresponding author. Study quality was evaluated using the modified Newcastle–Ottawa Scale (NOS) (17, 18), which assesses patient selection, standardized study protocol, and outcome measurement, with scores ranging from 1 to 7, where 7 represents the highest quality. Studies with NOS scores of 5 or above are considered of high quality. Data extracted for analysis included study characteristics (author, year, country, and design), diagnosis of the patients, patient details (number of the included patients, mean age at doxycycline exposure, routes of doxycycline administration, dosages, and treatment durations, mean follow-up durations, definition and validation of tooth discoloration, and the number of children who developed tooth discoloration in each study.

### Statistical analyses

Data for the incidence of tooth discoloration in children who received doxycycline in each study and their corresponding stand errors (SEs) were calculated from 95% CIs or *p* values, and were log-transformed to stabilize variance and normalize distribution (16). To assess heterogeneity, we used the Cochrane Q test and I² statistics (19), with I² < 25%, 25%–75%, and >70% indicating mild, moderate, and substantial heterogeneity among the included studies. A random-effects model was used to synthesize results while accounting for variability across studies (16). Subgroup analyses were performed to evaluate the predefined patient or study characteristics on the incidence, such as study country (Asian vs. European), design (prospective vs. retrospective), age group of the included children at doxycycline exposure (≤ or >8 years of age, or studies including children aged 0–18 years), routes of doxycycline administration (oral only or intravenous administration included), and methods for validation of tooth discoloration (direct observation by healthcare professionals or self-reported by the patients or their guardians). In addition, a univariate meta-regression analysis was also carried out to evaluate if the following characteristics may significantly affect the incidence of tooth discoloration after doxycycline exposure, such as the publication year of the study, sample size, mean age at doxycycline exposure, mean dosages of doxycycline, mean treatment durations, mean follow-up durations, and study quality scores by the modified NOS (16). Publication bias was assessed through funnel plots, visual asymmetry inspection, and Egger's regression test (20). A *p* value <0.05 indicates statistical significance. The statistical analyses were conducted using Stata software (version 12.0; Stata Corporation, College Station, TX, USA).

## Results

### Study identification

**Figure 1:**
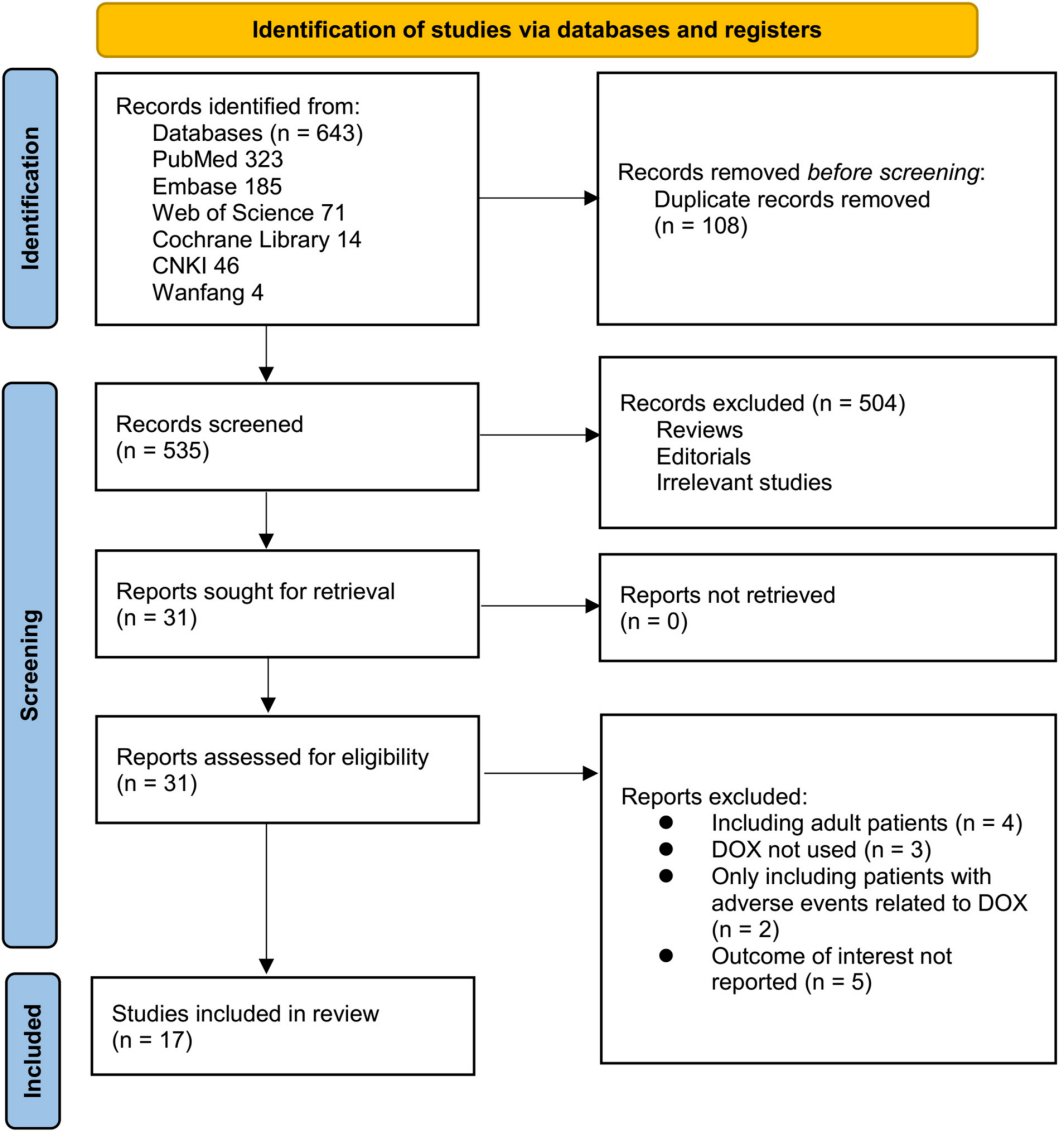
Flowchart of database search and study inclusion.

Figure 1 outlines the study selection process. Initially, 643 records were identified across three databases, with 108 duplicates removed. After title and abstract screening, 504 articles were excluded for not meeting the meta-analysis criteria. The full texts of the remaining 31 studies were independently reviewed by two authors, leading to the exclusion of 14 studies for reasons detailed in Figure 1. Ultimately, 17 studies were included in the quantitative analysis (21–37).

### Overview of the study characteristics

Table 1 presents a summary of the characteristics of the studies included in the meta-analysis. Overall, five prospective cohorts (23, 24, 30, 33, 36) and 12 retrospective cohorts (21, 22, 25–29, 31, 32, 34, 35, 37) were included in the meta-analysis. These studies were published between 1969 and 2025, and were performed in Italy, the United States, Israel, China, and Finland. Children who received doxycycline for various infectious diseases were included, including Rocky Mountain spotted fever (22, 25), atypical pneumonia and asthma (23), *Mycoplasma pneumoniae* pneumonia (24, 28, 30–37), Lyme disease (29), suspected infection of the central nervous system (26), inhalational anthrax, rickettsial infections, or ehrlichiosis (27). Overall, 1,025 children who received doxycycline were included. The age of the children at doxycycline exposure was within 8 years in eight studies (21–23, 25–27, 29, 37), over 8 years in three studies (28, 30, 36), and from 0 to 18 years in six studies (24, 31–35). Doxycycline was orally administered in 13 studies (21–25, 28–30, 33–37), intravenously in one study (32), and orally or intravenously in the other three studies (26, 27, 31). The mean dosages of doxycycline varied from 1.0 to 5.0 mg/kg/d. The treatment durations varied from 2 to 28 days. The mean follow-up durations were from 1 to 114 months after treatment. The detection and validation of tooth discoloration was via direct observation by healthcare professionals in 14 studies (21–28, 30, 33–37), and self-reported by the patients or their guardians in the other three studies (29, 31, 32). Overall, 15 children developed tooth discoloration during follow-up. The NOS scores ranged from four to seven, indicating moderate to high methodological and reporting quality (Table 2).

**Table 1:** Characteristics of the included studies.

Study	Country	Design	Diagnosis	No. of children included	Mean age	Route of DOX administration	Dosage of DOX	Treatment duration (days)	Mean follow-up duration (months)	Definition and validation of tooth discoloration	No. of children with tooth discoloration
Forti (21)	Italy	Retrospective	NR	25	4–55 day	Oral	2 mg/kg day 1, 1 mg/kg day 2 and afterward	6–17	12	Direct observation and fluorescence under Wood's light	1
Lochary (22)	USA	Retrospective	RMSF	10	4.3–8.3 years, mean: 5.1 years	Oral	15–100 mg bid	2–10	103	Direct observation	4
Volovitz (23)	Israel	Prospective	Atypical pneumonia and asthma	25	2–7.7 years, mean: 4.1 years	Oral	4 mg/kg bid on day 1, and 2 mg/kg qd on day 2 and afterward	10	76	Direct observation by pediatric dentist	0
He (24)	China	Prospective	RMPP	31	8–15 years, mean: 12.1 years	Oral	4 mg/kg bid on day 1, and 2 mg/kg bid on day 2 and afterward	5	NR	Direct observation at clinical visit	0
Todd (25)	USA	Retrospective	RMSF	58	0.2–7.9 years, mean: 4.5 years	Oral	2.3 mg/kg bid	1–10, mean: 7.3	64	Direct observation by dentists	0
Poyhonen (26)	Finland	Retrospective	Suspected CNS infection	38	0.6–7.9 years, mean: 4.7 years	Oral or IV	10 mg/kg/d for 2–3 d, then 5 mg/kg/d	2–28, mean: 12.5	114	Direct observation by dentists	0
Thompson (27)	USA	Retrospective	Inhalational anthrax, rickettsial infections, or ehrlichiosis	14	0.05–7.64, mean: 4.5 years	Oral or IV	1.69 mg/kg	NR	3	Direct observation	0
Pang (28)	China	Retrospective	SMPP	46	8.1–15.7 years, mean: 11.5 years	Oral	2.2 mg/kg bid	5–7	NR	Direct observation	0
Brown (29)	USA	Retrospective	Lyme disease	32	1.7–7 years, mean: 5.1 years	Oral	2.2 mg/kg bid	Mean: 12.9	NR	Self-reported by parents/guardians	2
Song (34)	China	Retrospective	RMPP	81	Mean: 10 years	Oral	2 mg/kg bid	10	NR	Direct observation	0
Li (30)	China	Prospective	MPP	50	8–12 years, mean: 10.1 years	Oral	2 mg/kg bid	7	NR	Direct observation	0
Zhou (36)	China	Prospective	MPP	103	8–12 years, mean: 10.1 years	Oral	2.2 mg/kg bid	7–10	NR	Direct observation	0
Lin (31)	China	Retrospective	RMPP	200	1.1–16 years, mean: 7.4 years	Oral (194) or IV (6)	NR	10	12	Self-reported by parents/guardians	8
Qiu (33)	China	Prospective	MPP	100	6–14 years, mean: 8.5 years	Oral	2 mg/kg bid	7–10	NR	Direct observation	0
Zhang (35)	China	Retrospective	RMPP	65	2.5–13.1 years, mean: 7.7 years	Oral	2 mg/kg bid	7–10, mean: 7.4	3	Direct observation	0
Ma (32)	China	Retrospective	RMPP	68	1–14 years, mean: 7.5 years	IV	2 mg/kg bid	10	1	Self-reported by parents/guardians	0
Li (37)	China	Retrospective	RMPP	79	< 8 years, mean: NR	Oral	2 mg/kg bid	10	5	Direct observation	0

CNS, central nervous system; DOX, doxycycline; IV, intravenous; MPP, *Mycoplasma pneumoniae* pneumonia; NR, not reported; RMPP, refractory *Mycoplasma pneumoniae* pneumonia; RMSF, Rocky Mountain spotted fever; SMPP, severe *Mycoplasma pneumoniae* pneumonia; USA, United States of America; bid, twice a day; q.d., once a day.

**Table 2:** Study quality evaluation via the modified Newcastle-Ottawa scale.

Study	Representativeness of the cohort	Confirmed use of DOX	Reporting study protocol and all pre-specified outcomes	Validated assessment of outcome	Enough long follow-up duration	Adequacy of follow-up of cohorts	Other bias	Overall quality
Forti (21)	0	1	0	1	1	1	1	5
Lochary (22)	0	1	0	1	1	1	1	5
Volovitz (23)	1	1	1	1	1	1	1	7
He (24)	1	1	1	1	0	1	1	6
Todd (25)	0	1	1	1	1	1	1	6
Poyhonen (26)	0	1	1	1	1	1	1	6
Thompson (27)	0	1	0	1	0	1	1	4
Pang (28)	1	1	0	1	0	1	1	5
Brown (29)	0	1	1	0	0	1	1	4
Song (34)	0	1	0	1	0	1	1	4
Li (30)	1	1	0	1	0	1	1	5
Zhou (36)	0	1	0	1	0	1	1	4
Lin (31)	0	1	1	0	1	1	1	5
Qiu (33)	1	1	0	1	0	1	1	5
Zhang (35)	0	1	1	1	0	1	1	5
Ma (32)	0	1	1	0	0	1	1	4
Li (37)	0	1	1	1	0	1	1	5

DOX, doxycycline.

### Incidence of tooth discoloration after doxycycline treatment in children

**Figure 2:**
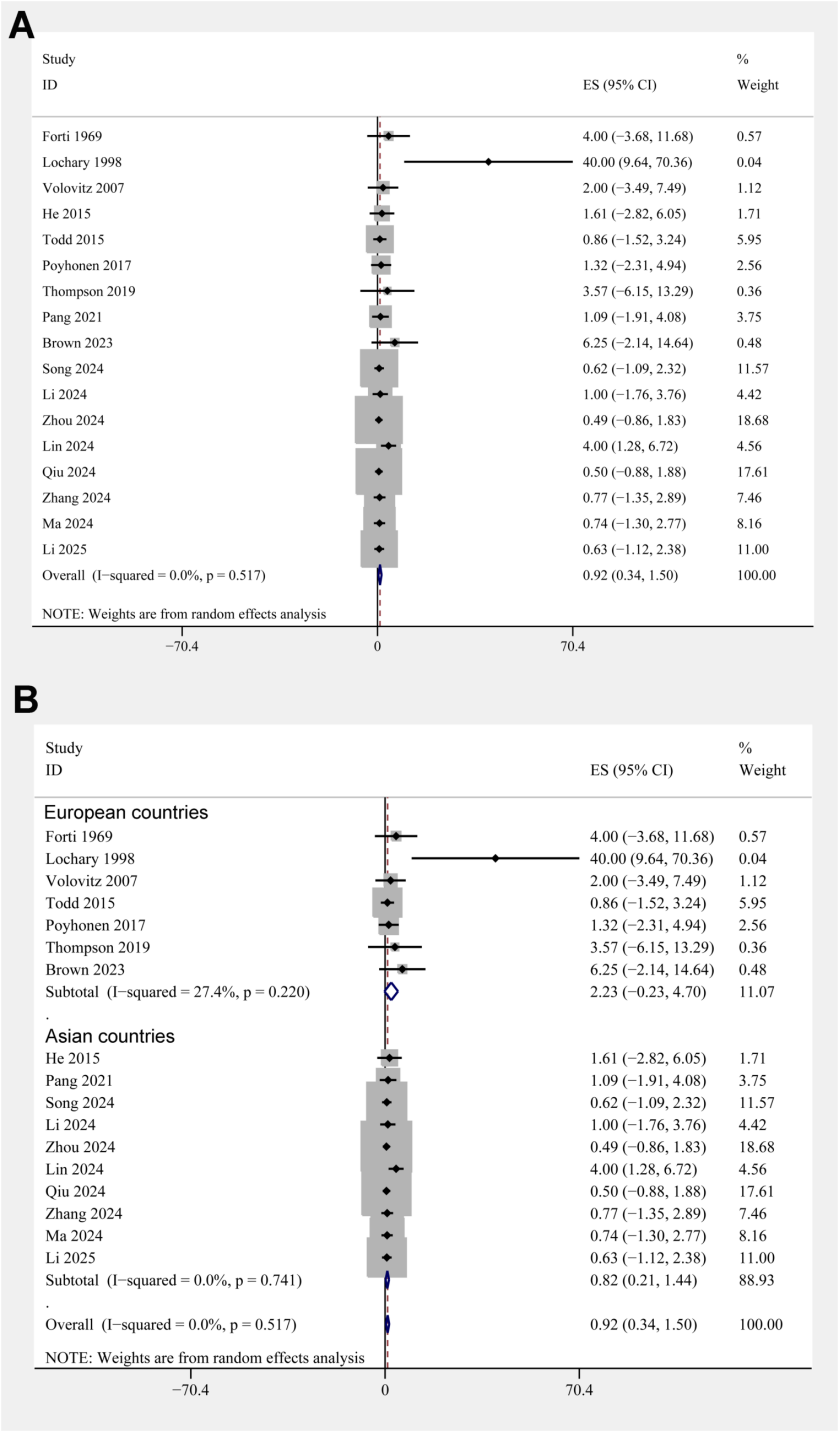
Forest plots for the meta-analysis of the incidence of tooth discoloration after doxycycline treatment in children. **(A)** Overall meta-analysis; and **(B)** subgroup analysis according to the country of the study.

**Figure 3:**
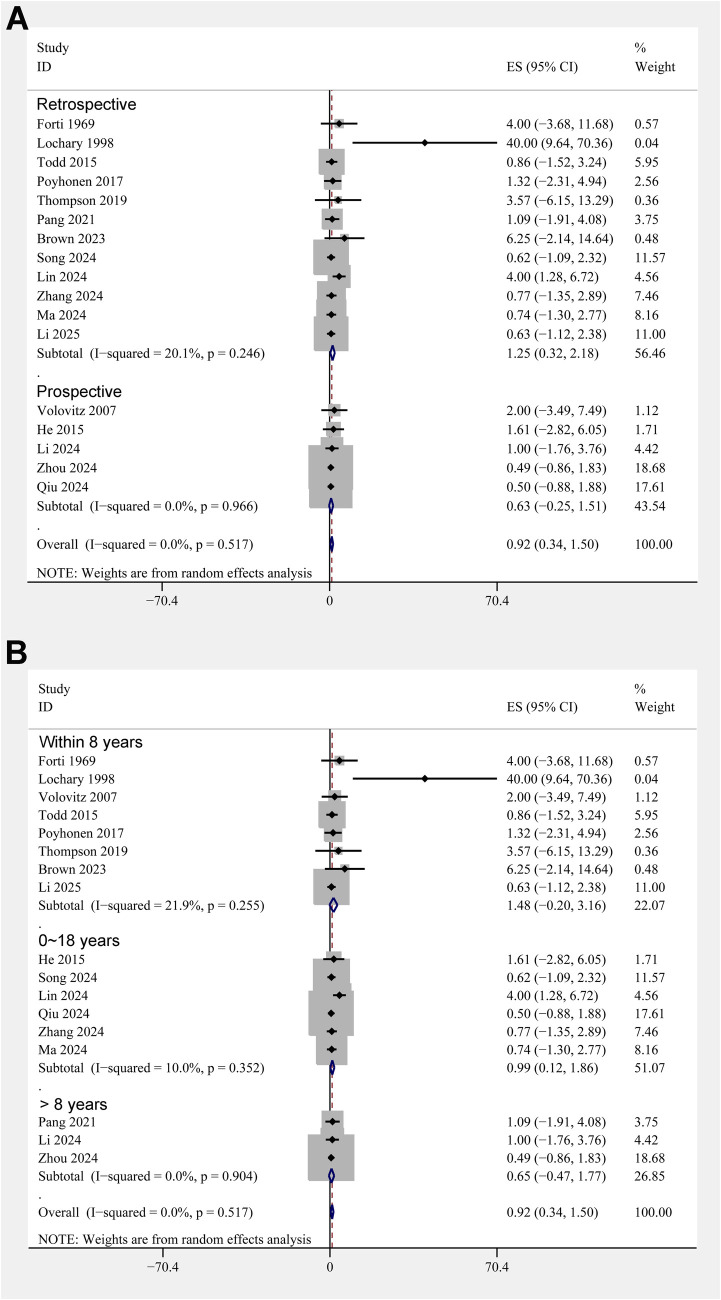
Forest plots for the subgroup analyses of the incidence of tooth discoloration after doxycycline treatment in children. **(A)** Subgroup analysis according to study design; and **(B)** subgroup analysis according to the age of doxycycline exposure.

**Figure 4:**
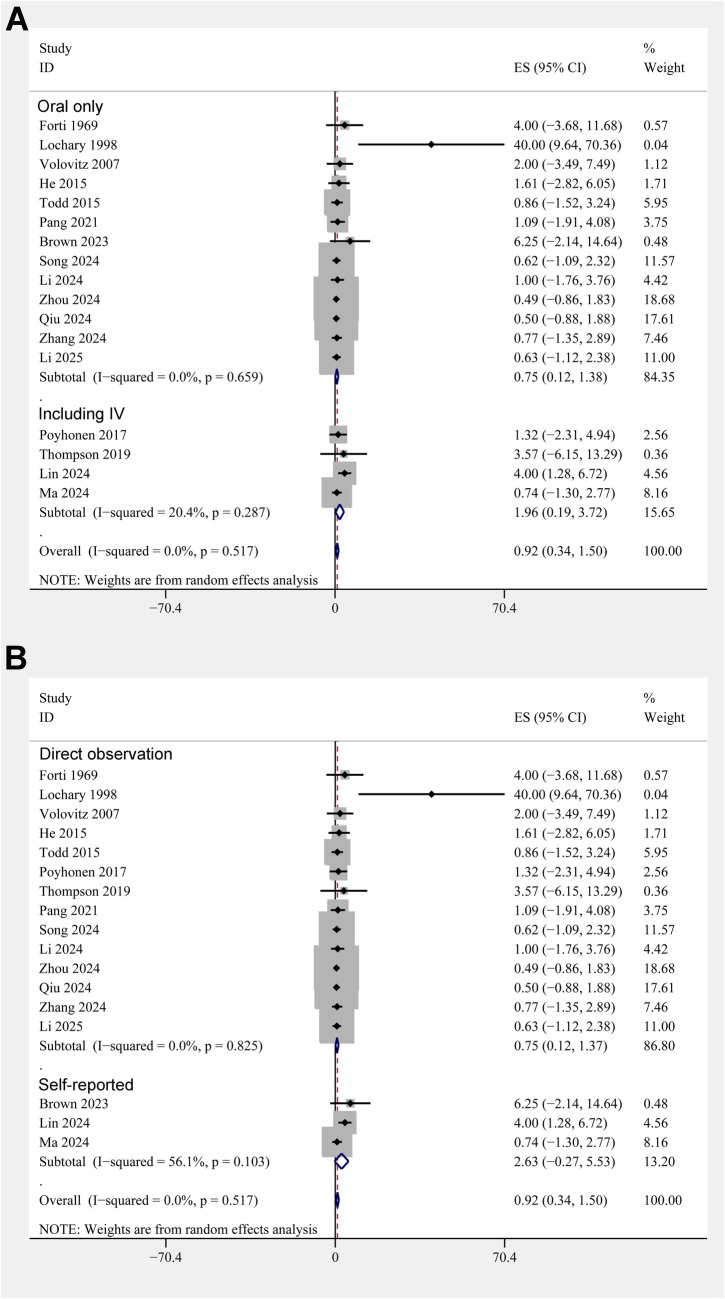
Forest plots for the subgroup analyses of the incidence of tooth discoloration after doxycycline treatment in children. **(A)** Subgroup analysis according to the routes of doxycycline administration; and **(B)** subgroup analysis according to the methods for validation of tooth discoloration.

The pooled results of 17 studies showed that the overall incidence of tooth discoloration after doxycycline treatment in children was 0.92% (95% CI: 0.34%–1.50%; Figure 2A) with no significant heterogeneity observed among the included studies (*p* for Cochrane Q test = 0.52, *I^2^* = 0%). Sensitivity analysis excluding the *Lochary 1998* study (22), which reported a higher incidence of tooth discoloration, showed a similar pooled incidence of 0.91% (95% CI: 0.33%–1.49%), with no change in heterogeneity (I² = 0%) (Supplementary Figure 1), supporting the robustness of the overall findings. Subsequent subgroup analyses showed that the incidence of tooth discoloration after doxycycline was not significantly different between children from European and Asian countries (2.23% vs. 0.82%, *p* for subgroup difference = 0.37; Figure 2B), in retrospective and prospective studies (1.25% vs. 0.63%, *p* for subgroup difference = 0.40; Figure 3A), and in children at doxycycline exposure within or over 8 years of age, or from 0 to 18 years (1.48%, 0.65%, vs. 0.99%, *p* for subgroup difference = 0.88; Figure 3B). Furthermore, consistent results were obtained for studies with doxycycline given only orally or those also including patients who received doxycycline intravenously (0.75% vs. 1.96%, *p* for subgroup difference = 0.82; Figure 4A), and in studies with tooth discoloration validated by direct observation and self-report (0.75% vs. 2.63%, *p* for subgroup difference = 0.15; Figure 4B). Finally, results of the univariate meta-regression analysis did not suggest that any of the variables may significantly influence the incidence of tooth discoloration after doxycycline treatment in children, including publication year of the study, sample size, mean age at doxycycline exposure, mean dosages of doxycycline, mean treatment durations, mean follow-up durations, or the modified NOS (*p* all >0.05; Table 3).

**Table 3:** Results of the univariate meta-regression analysis.

Variables	Incidence of tooth discoloration		
	Coefficient	95% CI	*p* values
Publication year	−0.068	−0.190–0.054	0.26
Sample size	0.0073	−0.0116–0.0262	0.42
Mean age (years)	−0.11	−0.39–0.18	0.43
Mean dose (mg/kg/d)	−0.10	−0.82–0.62	0.76
Mean treatment duration (days)	0.12	−0.34–0.57	0.59
Mean follow-up duration (months)	0.0087	−0.0179–0.0353	0.50
Quality scores	0.30	−0.63–1.22	0.50

CI, confidence interval.

### Publication bias

**Figure 5:**
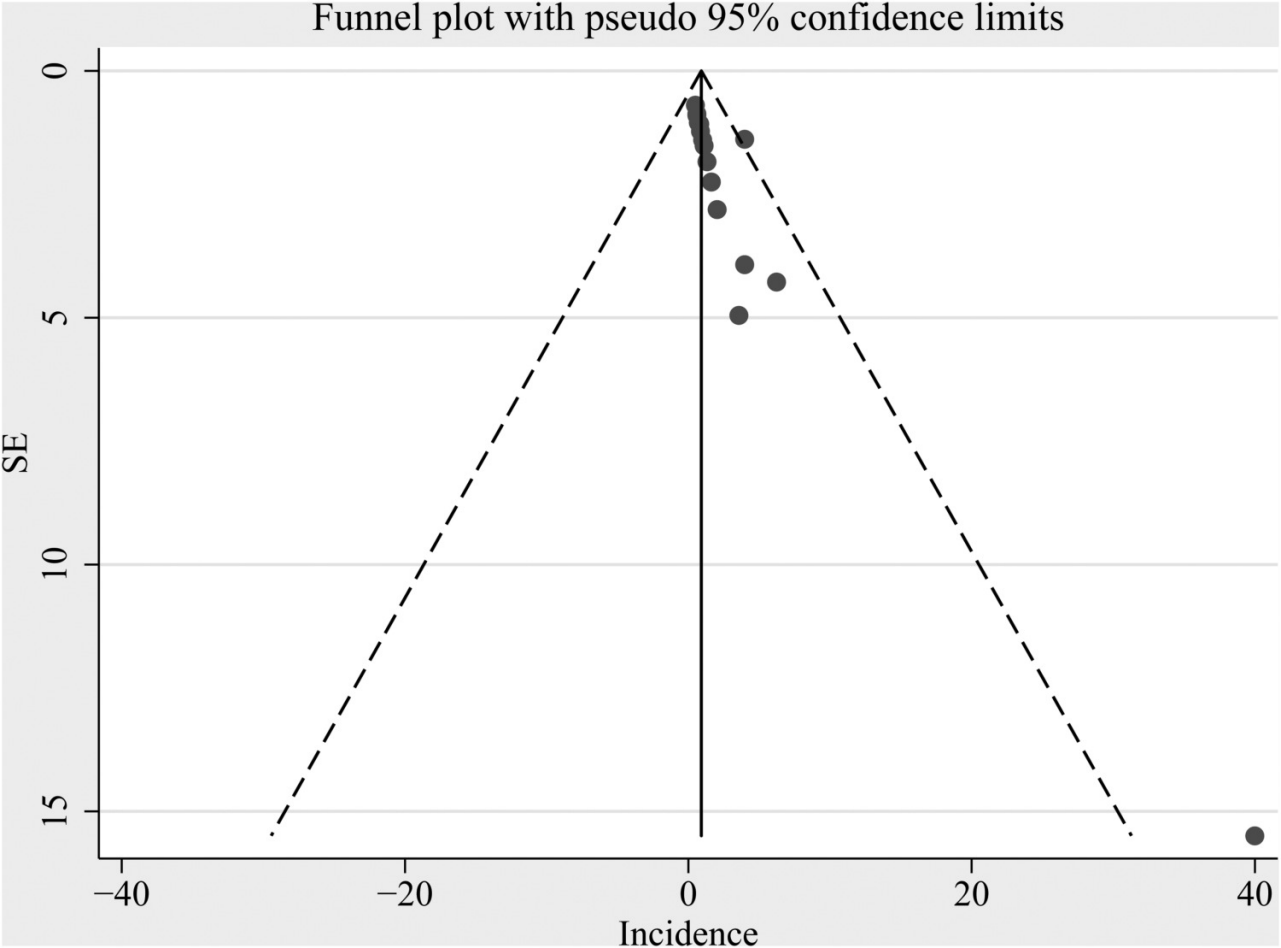
Funnel plot assessing potential publication bias underlying the meta-analysis of the incidence of tooth discoloration after doxycycline treatment in children.

Figure 5 displays funnel plots evaluating the publication bias underlying the meta-analysis of the incidence of tooth discoloration after doxycycline treatment in children. The plots seemed asymmetrical, suggesting the possible risk of publication bias. However, results of the Egger's regression test did not show a significant risk of publication bias (*p* = 0.15).

## Discussion

This meta-analysis synthesized evidence from 17 studies to estimate the incidence of tooth discoloration in children treated with doxycycline and to explore potential influencing factors. The pooled incidence of tooth discoloration was 0.92% (95% CI: 0.34%–1.50%), based on data from diverse pediatric populations and study designs. While this suggests a low overall incidence, the result should be interpreted cautiously due to variability in patient characteristics and outcome assessment across studies. Subgroup analyses revealed no significant differences in incidence by geographic region, study design, age group, administration route, or method of outcome assessment. Furthermore, univariate meta-regression analyses demonstrated that factors such as publication year, sample size, mean age at exposure, treatment duration, doxycycline dose, follow-up duration, and study quality scores did not significantly affect the reported incidence. These findings suggest that doxycycline may be safer in children, including those less than 8 years of age, than previously believed.

Our findings are consistent with and supported by emerging literature that challenges the traditional avoidance of doxycycline in young children. In a recent 2024 pharmacovigilance analysis based on the FDA Adverse Event Reporting System (FAERS), Qiao et al. evaluated adverse reports involving doxycycline, minocycline, and tigecycline in pediatric patients (38). Their findings demonstrated a low incidence of dental adverse events with doxycycline relative to other tetracycline derivatives, reinforcing its relative safety and suitability for use in young children when clinically warranted (38). Building on this, a more recent large-scale pharmacovigilance analysis by Zhang et al. evaluated 21,561 adverse event reports associated with tetracyclines and found that among children under 8 years of age, only doxycycline, minocycline, and omadacycline generated positive signals for tooth discoloration (39). Doxycycline had lower signal strength and fewer reported events than minocycline. Notably, the majority of reported cases in children were associated with short treatment durations (median onset: 60 days), and only one case in a 2.5-year-old child involved a 10-day course of doxycycline (39). These pharmacovigilance data further support the notion that the actual risk of tooth discoloration from doxycycline in children is very low, particularly when used appropriately and for short durations. Moreover, this study highlights that previous safety concerns may have been overstated due to historical high-dose exposures and less refined surveillance systems (39).

Our subgroup analyses provide further context for interpreting the main findings. The incidence of tooth discoloration did not significantly differ between children exposed before and after 8 years of age, suggesting that early age alone may not be a strong risk factor for discoloration under the typical clinical scenarios and treatment durations reported in these studies. Similarly, the route of administration (oral vs. oral/IV) and the method of outcome detection (direct observation vs. self-report) did not seem to significantly influence the incidence. Although one might expect IV administration or self-reported outcomes to increase variability or reporting bias, our results remained robust across these subgroups. Notably, the low heterogeneity (*I²*=0%) across studies supports the consistency of the findings and increases confidence in the pooled estimate. The very low heterogeneity (I² = 0%) likely reflects the uniformly low incidence observed, similar short-course dosing patterns, and the use of a binary, clinically observed endpoint across studies. However, this apparent consistency may also be influenced by under-ascertainment of mild cases, retrospective data capture, and small-study effects, potentially masking true between-study variability. Our subgroup and meta-regression analyses did not identify study-level modifiers, and although Egger's test was non-significant, the funnel plot suggested possible small-study or reporting bias. Therefore, the apparent “safety” signal should be interpreted cautiously, and prospective IPD-based studies with standardized dental assessments are warranted. On the other hand, meta-regression analyses did not identify any study-level factors that significantly modified the incidence of tooth discoloration. This includes treatment duration, doxycycline dose, and age at exposure, which were evaluated in the regression model but showed no statistically significant associations with the risk of discoloration. These findings suggest that short courses of doxycycline, as commonly used in pediatric infections, may not meaningfully increase the risk of tooth staining. The lack of associations in our meta-regression might also be explained by the limited variation in dosing and duration across studies, or by the inherent limitations of study-level analyses that cannot fully capture individual-level risk factors (40). While this strengthens the argument for a low overall incidence, it also underscores the need for patient-level data to better elucidate whether certain subgroups of children may still be at slightly elevated risk (40). In addition, a sensitivity analysis excluding the *Lochary 1998* study (22)—an early report with a relatively high incidence—yielded a nearly identical pooled estimate of 0.91% (95% CI: 0.33%–1.49%) and unchanged heterogeneity (I² = 0%). This consistency reinforces the reliability of our overall findings. The elevated rate in *Lochary 1998* (22) may reflect differences in follow-up duration, diagnostic thoroughness, or historical treatment practices.

Several strengths of this meta-analysis merit attention. First, we performed a comprehensive and up-to-date literature search across five databases, including both English and Chinese-language sources, to ensure broad and inclusive study representation. Second, the analysis included over 1,000 pediatric patients from diverse geographic regions and clinical contexts, enhancing generalizability. Third, the use of multiple subgroup and meta-regression analyses enabled a nuanced exploration of potential modifiers of risk. These analytical approaches, rarely used in prior reviews on this topic, add methodological rigor and depth to our findings. Nonetheless, there are limitations to consider. Most included studies were retrospective in nature, and therefore susceptible to recall and selection bias (41). In particular, retrospective designs relying on clinical records or parental recall may underreport or inconsistently document tooth discoloration, especially if it was mild or not the primary focus of the study (41). Second, although we included studies from both English and Chinese databases, we excluded articles in other languages, which may have led to a degree of publication bias despite Egger's test suggesting no significant risk (42). Third, as with most meta-analyses of observational studies, our subgroup and meta-regression analyses were based on aggregated study-level data rather than individual patient data. This ecological approach limits the ability to account for confounding variables and may mask associations present at the individual level (43). In addition, follow-up durations varied considerably among the studies, and some were relatively short, potentially missing delayed or subtle manifestations of dental changes. Finally, although the included studies varied in patient age, clinical indications, and outcome assessment methods, our subgroup and meta-regression analyses consistently showed no significant differences in the incidence of tooth discoloration across these factors. Due to the lack of individual-level data, we were unable to directly assess risk during the specific window of permanent tooth formation. As an alternative, we stratified subgroup analyses using study-level median or mean age at exposure (e.g., ≤8 years vs. >8 years) to approximate the risk window. While this approach offers practical value, it may not precisely reflect individual variation in tooth development. Moreover, although minor shifts in the timing of permanent tooth eruption may have occurred across populations or over time, current evidence suggests that the general timeline for eruption has remained relatively stable in recent decades. Future individual participant data (IPD)-level studies would allow for more accurate stratification by dental developmental stage. In addition, we also recognize that tooth discoloration may involve not only enamel but also underlying dentin, and that the risk may be influenced by the timing of tooth bud formation—ranging from gestation through infancy for deciduous teeth, and from infancy through approximately 7–8 years for permanent teeth (44). However, due to the unavailability of IPD, we were unable to separately analyze outcomes in children exposed during these critical developmental periods. Instead, age-based stratification was performed at the study level using median or mean age, which may not precisely align with the biological windows of tooth bud formation. The overall statistical heterogeneity was low (I² = 0%), suggesting that the pooled estimate is stable despite variation in study characteristics. Nonetheless, we acknowledge that differences in study design and assessment approaches may introduce unmeasured bias and should be interpreted with caution. Finally, it is also important to note that while our analysis focused on tooth discoloration, doxycycline may theoretically carry a risk of enamel hypoplasia, particularly when administered during early stages of tooth development (26). However, none of the included studies reported enamel hypoplasia as a separate outcome, and most assessments relied on direct clinical observation or caregiver report, which are more likely to detect visible discoloration than structural enamel defects. As such, our findings may not capture the full spectrum of potential dental effects associated with doxycycline, underscoring the need for future studies with comprehensive dental evaluations.

From a clinical standpoint, the findings of this meta-analysis have important implications. In conditions where doxycycline is the most effective or recommended therapy—such as rickettsial infections, atypical pneumonia, or macrolide-resistant *Mycoplasma pneumoniae*—its use in children, including those under 8 years of age, should not be automatically precluded due to concerns about tooth discoloration alone. The low incidence reported here, together with supporting evidence from recent prospective and pharmacovigilance studies (38, 39), suggests that the historical restriction on pediatric doxycycline use may be overly cautious. This is consistent with updated CDC recommendations, which now endorse doxycycline for children of all ages in specific life-threatening infections (45, 46). Future research should focus on prospective, controlled studies with standardized dental assessments and sufficient follow-up to capture any late-onset effects. Ideally, studies should incorporate patient-level data to enable more precise risk stratification based on age, dosing, cumulative exposure, genetic factors, and dental development stage. Moreover, although our meta-analysis focused exclusively on doxycycline, we acknowledge that direct comparisons with other tetracyclines (e.g., tetracycline or minocycline) could offer valuable insight. However, such analyses were not feasible due to the lack of comparable incidence data or direct comparative studies. Future meta-analyses or head-to-head observational studies are warranted to better contextualize the relative dental risks across different tetracycline-class agents. In addition, exploration of parent and patient perceptions of dental side effects could provide insight into the psychosocial impact of tooth discoloration and inform shared decision-making in clinical practice.

## Conclusions

In conclusion, this meta-analysis demonstrates that the incidence of tooth discoloration following doxycycline treatment in children is low, and not significantly affected by patient age, administration route, or study design. These findings support a re-evaluation of long-standing prescribing restrictions and provide evidence to guide safer, more informed use of doxycycline in pediatric care. With appropriate clinical judgment, doxycycline may be a reasonable therapeutic option even in children under 8 years of age, particularly when the benefits clearly outweigh the risks.

## Data Availability

The original contributions presented in the study are included in the article/Supplementary Material, further inquiries can be directed to the corresponding author.
