# Supplementary material for: Incidence and influencing factors of tooth discoloration in children using doxycycline: a meta-analysis
Source: Front Pediatr. 2025 Aug 22;13:1644231. doi: 10.3389/fped.2025.1644231 (PMC12411547; doi:10.3389/fped.2025.1644231)
Supplement: Supplementary file 1 [file Datasheet1.pdf]

PubMed

((doxycycline[MeSH Terms] OR tetracycline[MeSH Terms] OR doxycycline OR tetracycline) AND ("tooth"[MeSH Terms] OR "teeth"[MeSH Terms] OR "dental"[All Fields] OR tooth OR teeth OR dental) AND ("staining"[MeSH Terms] OR "discoloration"[All Fields] OR "pigmentation"[MeSH Terms] OR staining OR discoloration OR pigmentation OR "events"[All Fields]) AND ("child"[MeSH Terms] OR "children"[All Fields] OR "pediatric"[All Fields] OR "adolescents"[MeSH Terms] OR child OR children OR pediatric OR adolescents))

Embase

('doxycycline'/exp OR 'tetracycline'/exp OR doxycycline OR tetracycline) AND ('tooth'/exp OR 'teeth'/exp OR 'dental'/exp OR tooth OR teeth OR dental) AND ('staining'/exp OR 'discoloration'/exp OR 'pigmentation'/exp OR staining OR discoloration OR pigmentation OR events) AND ('child'/exp OR 'children'/exp OR 'pediatric'/exp OR 'adolescents'/exp OR child OR children OR pediatric OR adolescents) AND [embase]/lim

Web of Science

((doxycycline OR tetracycline) AND (tooth OR teeth OR dental) AND (staining OR discoloration OR pigmentation OR events) AND (child OR children OR pediatric OR adolescents))

Cochrane Library

(doxycycline OR tetracycline) AND (tooth OR teeth OR dental) AND (staining OR discoloration OR pigmentation OR events) AND (child OR children OR pediatric OR

adolescents)

<u>CNKI</u>

多西环素[篇名] AND 儿童 AND 牙齿

<u>Wanfang</u>

全部:(多西环素) and 全部:(儿童) and 全部:(牙齿)
