# Supplementary figures and images for: Incidence and influencing factors of tooth discoloration in children using doxycycline: a meta-analysis
Source: Front Pediatr. 2025 Aug 22;13:1644231. doi: 10.3389/fped.2025.1644231 (PMC12411547; doi:10.3389/fped.2025.1644231)

**Supplemental Figure 1** Forest plots for the sensitivity analysis excluding study by Lochary 1998


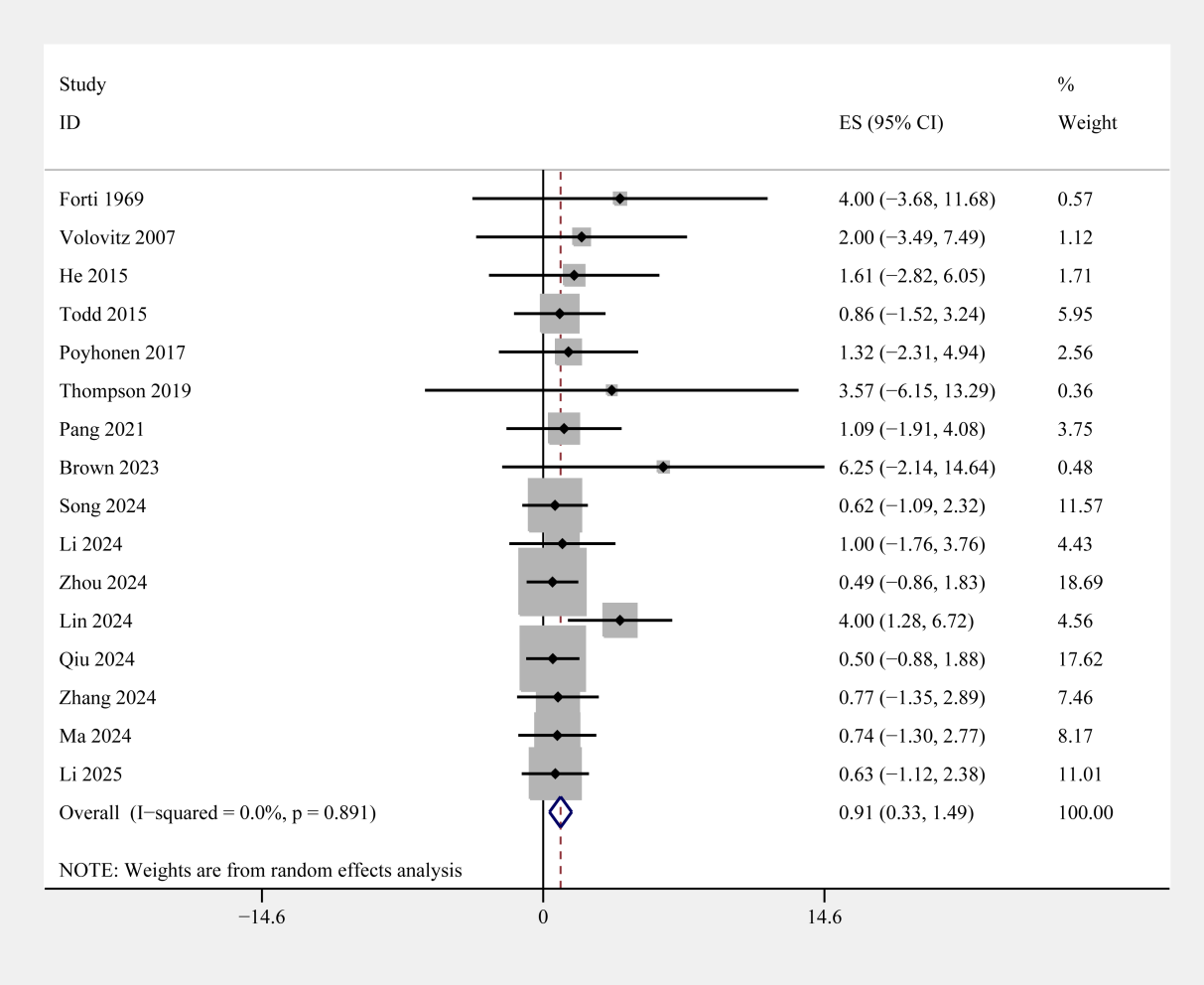

Supplement: Supplementary file 2 [file Supplementaryfile1.docx]
